# Cerebral venous sinus thrombosis and skull base osteomyelitis as manifestations of cat‐scratch disease in a pediatric patient: A case report and literature review

**DOI:** 10.1002/ccr3.7561

**Published:** 2023-06-22

**Authors:** Ayodeji Otufowora, Christine Lee, Aneeb Mohideen, Grace DeSena, Charlene Pringle, Freddie Guyer, Priya Sharma, Torrey Baines, Silvana Carr

**Affiliations:** ^1^ Department of Pediatrics College of Medicine, University of Florida Gainesville Florida USA; ^2^ Department of Radiology College of Medicine, University of Florida Gainesville Florida USA

**Keywords:** *Bartonella henselae*, cat‐scratch disease, cerebral venous sinus thrombosis, headache, skull base osteomyelitis

## Abstract

Cat‐scratch disease (CSD) is caused by *Bartonella henselae* and usually presents with regional lymphadenopathy. Skull base osteomyelitis and cerebral venous sinus thrombosis are rarely reported, particularly in immunocompetent children. CSD should be considered in the differential diagnosis of any patient with persistent headaches in the setting of cat exposure.

## Graphical abstract

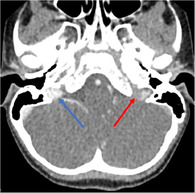

## 1 INTRODUCTION

*Bartonella henselae* represents the most frequent etiologic agent of cat‐scratch disease (CSD), a common childhood vector‐borne zoonotic disease.^1^ Domestic cats are the natural reservoir and vectors of *B. henselae*, but other animals, such as dogs, have been implicated as possible disease reservoirs.^2^ *Bartonella* spp. are facultative, fastidious intracellular gram‐negative bacillus.^3^ Infection is transmitted from cats to humans by a scratch or a bite and is caused by the presence of the bacterium on claws or in the oral cavity.^4^ In the immunocompetent host, approximately 90% of cases are limited to a subacute, solitary, or regional lymphadenopathy, and in one‐half of the cases, CSD is associated with systemic symptoms, such as low‐grade fever, headache, poor appetite, and fatigue that spontaneously resolves by itself within 2–4 months in most cases.^5^ The treatment of *Bartonella* infections with antibiotics depends on the clinical presentation of the disease and the immune status of the patient. We report a case of a child who developed a right submandibular (RSM) lymphadenopathy after being scratched by a cat on the right side of her face. Our patient then developed left parieto‐occipital headache, emesis, and photophobia; symptoms that were found to be secondary to left‐sided skull base osteomyelitis complicated by venous sinus thrombosis due to *B. henselae* infection.

## 2 CASE PRESENTATION

A previously healthy 3‐year‐old female with no significant past medical history presents to her pediatrician with a history of intermittent fever (highest temperature of 39.4°C), lethargy, RSM lymphadenopathy, and left parieto‐occipital pain 10 days after being scratched on the right cheek by her pet kitten. She started oral cephalexin (40 mg/kg/day BID × 10 days) for presumed bacterial lymphadenitis. On Day 7 of oral cephalexin, the patient presents to our hospital emergency department (ED) due to lack of symptomatic improvement despite antibiotic therapy. She was subsequently admitted to the pediatric floor.

At admission, the physical examination revealed a temporal temperature of 37.2°C, a blood pressure of 111/66 mmHg, a heart rate of 122 beats per minute, and a respiratory rate of 22 breaths per minute. A 2.0 cm RSM mildly tender, mobile, indurated but nonsuppurative lymph node was appreciated. There were no clinical signs of tenderness, swelling, or skin erythema on her head, and physical examination was otherwise within the normal limits (WNL). She was started on empirical therapy with IV clindamycin and oral azithromycin for presumed bacterial and *B. henselae* lymphadenitis. The initial laboratory tests on admission were significant for elevated inflammatory markers, but otherwise unremarkable (Table 1).

**TABLE 1:** Initial laboratory test results on admission.

Laboratory test	Result	Reference range and units
CBC/differential		
White blood cell	14.0	6.0–17.5 thou/cumm
Neutrophils	71.3	35.0%–85.0%
Lymphocytes	21.5	25.0%–65.0%
Monocytes	5.7	2.0%–10.0%
Eosinophils	1.1	0.0%–8.0%
Basophils	0.4	0.0%–2.0%
Red blood cell	4.16	3.7–5.5 × 10^6^/μL
Hemoglobin	11.6	10.5–13.5 G/dL
Hematocrit	34.0	33.0%–39.0%
Platelet count	581	150–450 thou/cumm
Basic metabolic panel		
Sodium	137	136–145 mmol/L
Potassium	4.2	3.3–5.1 mmol/L
Chloride	102	9.8–107 mmol/L
CO_2_	21	22–30 mmol/L
Urea nitrogen	9	6–21 mg/dL
Creatinine	0.35	0.20–0.43 mg/dL
BUN/creatinine ratio	25.7	CALC
Glucose	80	65–99 mg/dL
Calcium	10.0	8.4–10.2 mg/dL
Inflammatory markers		
CRP	104.11	0.00–5.00 mg/L
ESR	71	0–20 mm/h

Given a positive history of a cat scratch and a right SM lymphadenitis on physical examination, *B. henselae* IgM and IgG serology was ordered, and the patient was discharged home the next day on oral clindamycin (15 mg/kg/day TID) for an additional 6 days and azithromycin (5 mg/kg daily–single daily dose) for an additional 4 days.

**FIGURE 1:**
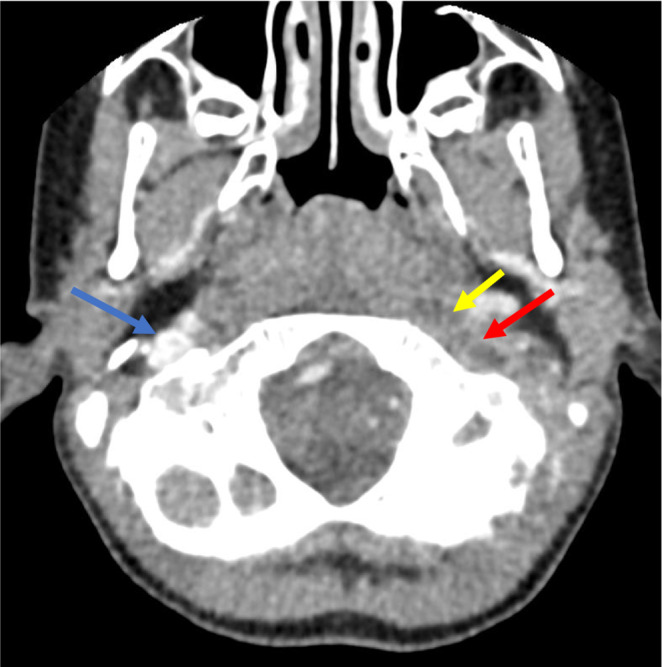
Axial contrast‐enhanced CT of the neck demonstrating normal contrast enhancement of the upper right internal jugular vein (blue arrow). Note the lack of contrast enhancement of the upper left internal jugular vein (red arrow). Central hypodensity within this structure is compatible with internal thrombus. Adjacent retropharyngeal edema is marked by the yellow arrow.

**FIGURE 2:**
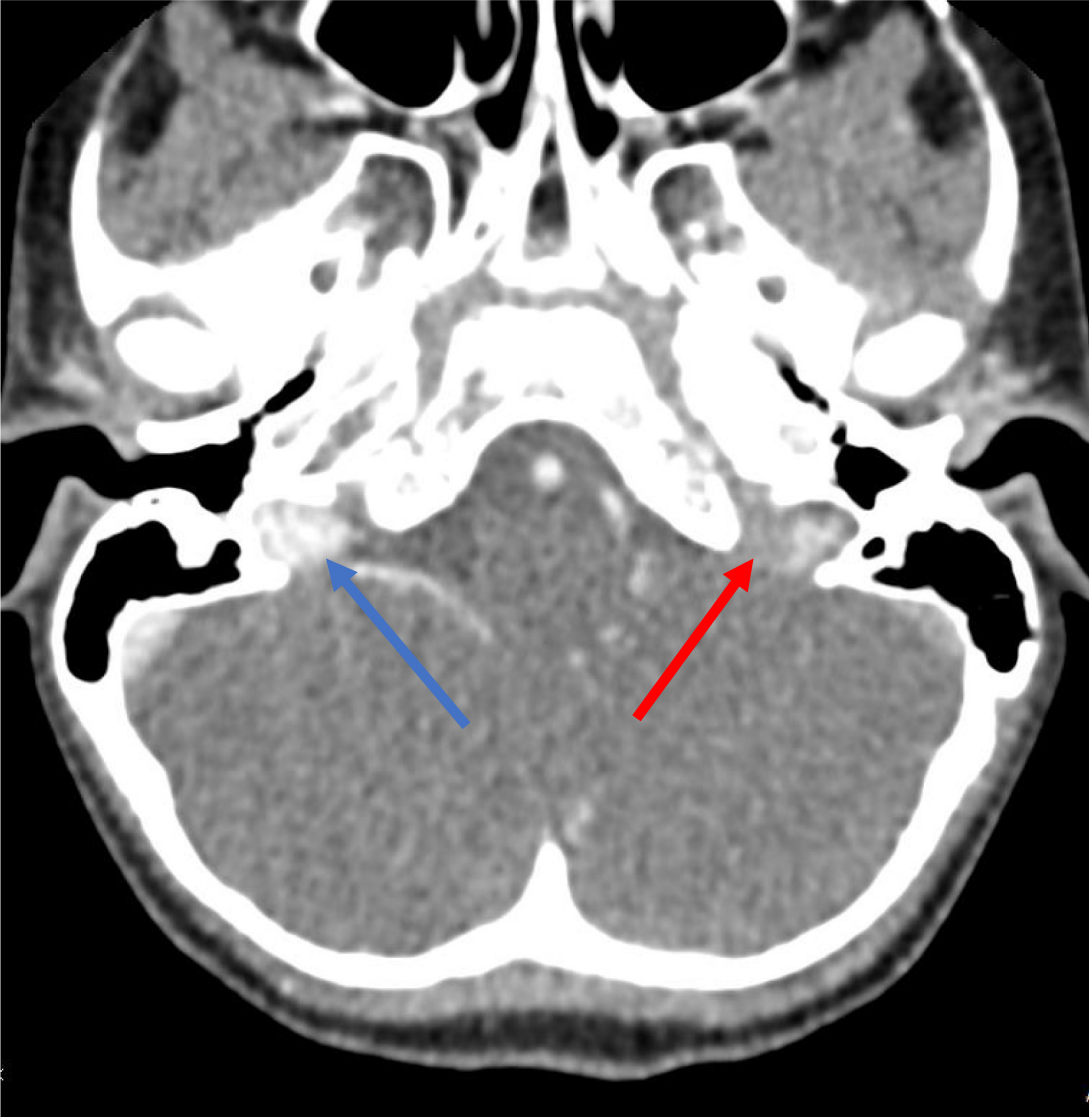
Axial contrast‐enhanced CT of the neck demonstrating normal contrast enhancement at the level of the right jugular bulb (blue arrow). Note the lack of contrast enhancement of the left jugular bulb (red arrow).

Six days after hospital discharge, the child presents at her PCP's office with worsening left occipital pain and emesis. A head computerized tomography (CT) with contrast was ordered and revealed the presence of right‐sided SM lymphadenitis and sub‐acute thrombophlebitis in the left sigmoid sinus extending to the jugular bulb with an adjacent small suppurative left retropharyngeal space lymph node without associated abscess (Figures 1 and 2).

The patient was subsequently admitted to the pediatric intensive care unit (PICU) to start anti‐coagulation therapy, as recommended by the pediatric neurology and hematology services. At admission, physical examination revealed a temporal temperature of 36.8°C, a blood pressure of 133/92 mm Hg, a heart rate of 138 beats per minute, and a respiratory rate of 24 breaths per minute. Physical examination was remarkable for a tired and ill‐appearing child with a non‐tender, mobile, right jugulodigastric node measuring 1.5 cm in the short axis, associated with the previously described right SM node that had decreased in size to 1.5 cm and remained mildly tender, mobile and without skin discoloration or drainage. Otherwise, the physical examination was WNL including the neurological examination.

The initial laboratory tests in the pediatric ICU (Table 2) were significant for elevated inflammatory markers and abnormal coagulation studies.

**TABLE 2:** Initial laboratory test results in the pediatric ICU.

Laboratory test	Result	Reference range and units
CBC/differential		
White blood cell	14.8 × 10^3^	6.0–17.5 × 10^3^/mL
Neutrophils	77	35.0%–85.0%
Lymphocytes	17.1	25.0%–65.0%
Monocytes	4.8	2.0%–10.0%
Eosinophils	0.6	0.0%–8.0%
Basophils	0.5	0.0%–2.0%
Red blood cell	4.33	3.7–5.5 × 10^6^/μL
Hemoglobin	12.4	10.5–13.5 g/dL
Hematocrit	34.9	33.0–39.0%
Platelet count	618	150–450 thou/cumm
Inflammatory markers		
CRP	46.49	0.00–5.00 mg/L
ESR	80	0–20 mm/h
Transaminases		
AST	20	0–37 IU/L
ALT	4	0–35 IU/L
Coagulation factors		
Prothrombin time	13.8	9.1–13.5 s
aPTT	38	15–38 s
INR	1.2	0.8–1.1
Fibrinogen	576	173–454 mg/dL

**FIGURE 3:**
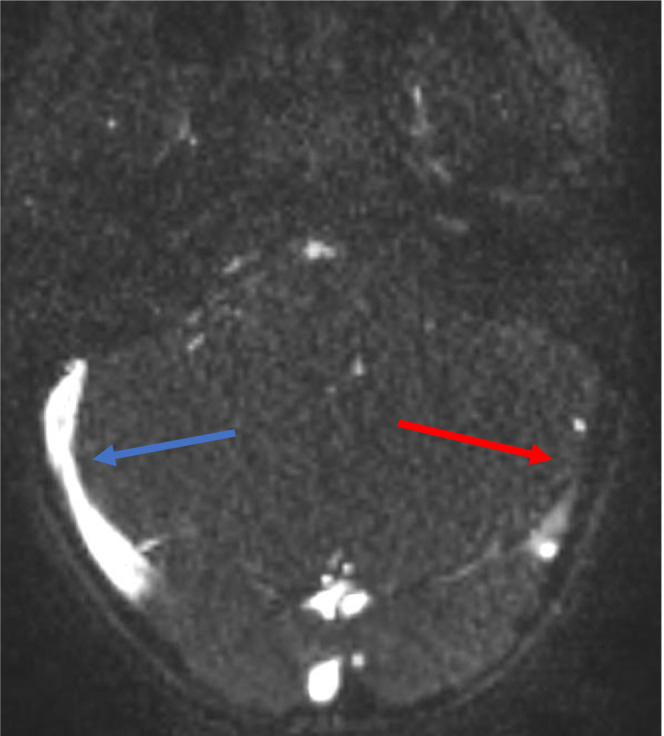
Selected axial image from contrast‐enhanced 3D MRV of the head demonstrating normal contrast enhancement of the right transverse sinus (blue arrow). Note the lack of contrast enhancement of the left transverse sinus (red arrow).

**FIGURE 4:**
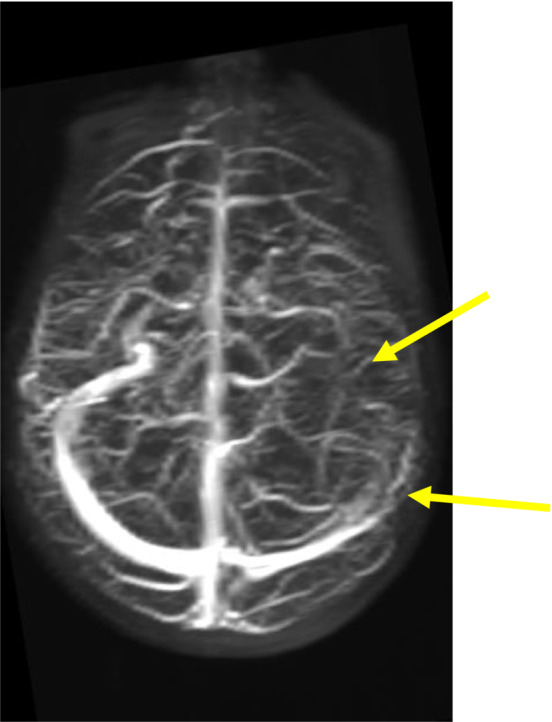
Sagittal oblique MRV time of flight maximum intensity projection after the administration of contrast demonstrates a lack of opacification of the left distal transverse and sigmoid sinuses (yellow arrows).

**FIGURE 5:**
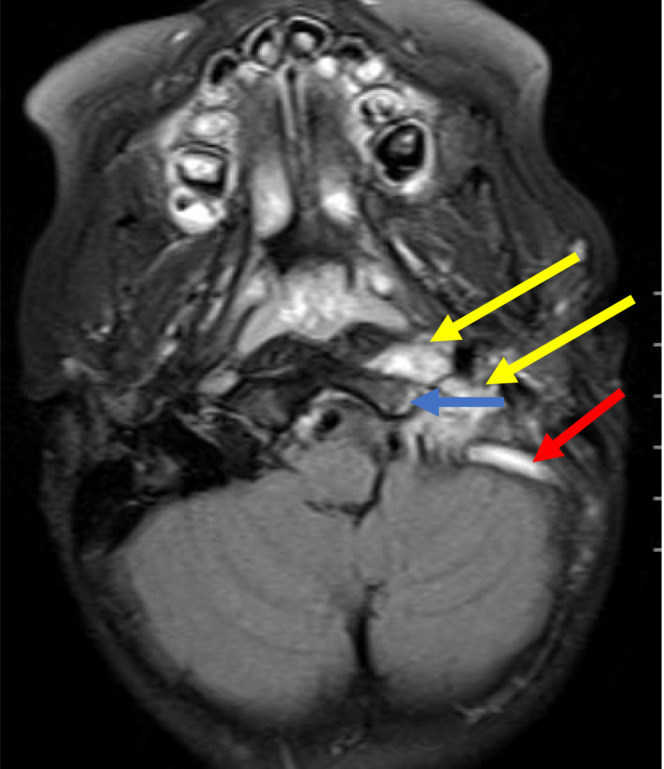
Axial T2 FLAIR image demonstrating abnormal soft tissue edema and associated retropharyngeal lymphadenopathy (yellow arrow). Associated bone marrow edema involving the left occipital bone (blue arrow). Also, note the lack of T2 flow void in the sigmoid sinus on the left–this is compatible with a thrombus (red arrow).

Bartonella serology result from previous hospitalization revealed an elevated *B. henselae* IgG (>1:1024; negative: < 1:64) and IgM (1:128; negative: < 1:16). Eye examination, chest x‐ray and complete abdominal ultrasound were WNL. A brain MRI and MRV were completed (Figures 3, 4, 5), and results confirmed the previous CT findings and revealed left occipital bone and clivus osteomyelitis without lytic lesions and dural sinus thrombophlebitis involving the left distal transverse sinus, left sigmoid sinus, left jugular bulb, and upper internal jugular vein; there was no intracranial abscess (Figure 1).

Intravenous trimethoprim‐sulfamethoxazole (20 mg/kg/day q 8 h) and rifampin (20 mg/kg/day q 24 h) were started to treat left occipital osteomyelitis presumed caused by CSD as per the pediatric infectious diseases service recommendations. The blood culture did not grow any micro‐organisms and blood *B. henselae* PCR, performed 18 days after SM lymphadenopathy onset, was negative. The patient remained afebrile with the resolution of her left occipital pain and was discharged home on Day 11 of hospital admission on IV trimethoprim‐sulfamethoxazole and rifampin through a central venous line and subcutaneous enoxaparin.

At the follow‐up outpatient visit 7 days after discharge, the patient remained afebrile with improved appetite; she was more active and without any complaints of pain. Follow‐up ESR and CRP on Day 20 of antibiotic therapy were WNL. On Day 33 of IV antibiotic therapy, a brain MRV was performed showing complete resolution of the left distal transverse and sigmoid sinuses thrombosis and partial resolution of the upper left internal jugular vein thrombus; contrast enhancement over the left occipital condyle and clivus was unchanged. Subcutaneous enoxaparin was discontinued by pediatric hematology. IV antibiotic therapy was switched to oral rifampin (20 mg/kg/day BID) and trimethoprim‐sulfamethoxazole (15 mg/kg/day TID). Follow‐up *B. henselae* serology on Day 36 of antibiotic therapy revealed negative IgM (<1:16) and IgG (titer of 1:1024). The patient completed a total of 9 weeks of combined IV and oral antibiotic therapy and follow‐up *B. henselae* serology at therapy completion revealed negative IgM (<1:16) and a lower IgG titer of 1:512.

At her 3‐month follow‐up visit after completing treatment, the patient remained asymptomatic and was back to her baseline clinical status. She did not complain of a headache. On physical examination, there was complete resolution of the previous right SM lymphadenopathy, and physical examination was otherwise WNL. An immunological workup was not completed because her mother refused further blood tests.

## 3 DISCUSSION

CSD is the most common and typical manifestation of *B. henselae* infection worldwide. In the immunocompetent host, the disease is limited to a subacute, solitary, or regional lymphadenopathy frequently associated with a low‐grade fever, headache, poor appetite, and fatigue that spontaneously resolves in a few weeks. Lymphadenopathy occurs 1–3 weeks after a scratch, bite, or other contact with an infected kitten or cat. One or more 3‐ to 5‐mm red or brown nontender papules develop in most patients at the site of inoculation 3–30 days after the infectious contact. These cutaneous papules usually progress to erythematous, vesicular, and papular crusted lesions in 1–3 weeks. Affected nodes in order of location frequency are the upper extremities (46%, axillary and epitrochlear nodes); the neck and jaw region (26%, cervical and submandibular nodes); the groin (17.5%, femoral and inguinal); the preauricular (7%); and the clavicular (2%) regions. In 10%–20% of cases, more than one region is affected.^2^ Lymphadenopathy is moderately tender, although some nodes are non‐tender. The skin over the nodes is often warm and erythematous. The lymph node enlargement usually resolves within 9 weeks but may last up to 6–12 months. Histologic examination reveals granulomas with multiple micro‐abscesses. Up to 10% of lesions progress with spontaneous drainage.^3^

Infection dissemination to the liver, spleen, eye, bone, and central nervous system, once called an atypical form of CSD, may occur in 5%–20% of cases, mainly in immunocompromised patients, and may lead to life‐threatening complications.^6^ Lymphadenopathy may precede the development of systemic infection through a symptom‐free period.^7^ Bartonella osteoarticular infections are well‐recognized yet uncommon, and even rarer is skull involvement.^8^ In the literature, bone involvement during CSD accounts for 0.17%–0.27% of all CSD cases and affects mostly children^2^, ^9^; 75% of cases are unifocal and affects the axial skeleton, such as the skull, sternum, ribs, spine, and pelvis.^8^, ^10^

In a literature review prior to 2007, Hajjaji et al.^8^ identified 36 cases of pediatric Bartonella osteomyelitis of which only 4 cases were skull‐based osteomyelitis. Dona and colleagues^11^ in 2018 used the same search terms as Hajjaji et al.^8^ (“Bartonella,” “bone,” “osteolytic,” “osteomyelitis,” and “cat‐scratch”) and articles indexed in PubMed, Embase, and Google scholar, and identified 51 cases Bartonella osteomyelitis, of which 10 were skull‐based. Of note, Rafee and English^12^ also reported a case of Bartonella skull osteomyelitis affecting the left frontal bone in a pediatric patient in 2018. Using the same search terms and same databases used by Dona and colleagues,^11^ we found no reported cases of skull‐base osteomyelitis in a pediatric population since 2019. Thus, to our knowledge, 12 cases of pediatric Bartonella skull‐based osteomyelitis (including this study) have been reported in the literature.

Unlike *S. aureus* osteomyelitis, signs such as rubor and calor are extremely rare of CSD bone infection, and primary osteomyelitis with no other systemic manifestation is the most common presentation.^8^, ^11^ Other musculoskeletal (MSK) manifestations of CSD include myalgia, arthropathy (arthralgia and/or arthritis) and tendinitis. Although osteomyelitis is the most well‐known MSK manifestation of CSD, in a large cohort of 913 CSD healthy patients, the incidence of myalgia and arthropathy were higher than that for osteomyelitis (5.8% and 5.5% vs. 0.2%, respectively). In the same study, myalgia was negatively associated with head and neck location of lymphadenopathy for unclear reasons. Arthropathy was significantly more common in female patients aged >20 years, was associated with erythema nodosum, and occurred early in the course of the infection with approximately 50% of the involved joints being the weight‐bearing joints.^10^

The estimated incidence of cerebral venous sinus thrombosis (CVST) in children varies between 0.4–0.7/100,000.^13^ Dehydration and systemic and local infections of the head and neck like mastoiditis and meningitis are the most common conditions associated with CVST among previously healthy children.^14^, ^15^ Clinical signs and symptoms of CSVT varies according to the age and underlying acute or chronic illness. Outside of the neonatal period, CSVT usually presents with depressed mental status, headache, and vomiting. Other signs and symptoms will depend on the underlying provoking illness.^16^, ^17^, ^18^, ^19^ In the case of our patient, CSVT contributed to her persistent emesis and left head pain requiring initial PICU admission. The triad of symptoms—progressive unremitting headache, altered mental status, and vomiting should prompt consideration of a diagnosis of CSVT and neuroimaging evaluation of children with *B. henseale* infection.

CSD infection disseminates in three ways: hematogenous, lymphatic, or contiguous.^20^ In cases of osteomyelitis, only 39.3% of lymph nodes were contiguous or in drainage area of the bone infected site, indicating potentially more hematogenous dissemination of CSD in children.^11^ In our case, it is possible that hematogenous seeding of the occipital bone may have occurred during the early infection, but we are unable to confirm hematogenous spread of Bartonella due to her negative blood PCR. The gold standard test modality for CSVT is an MRI with a magnetic resonance venogram (MRV).^21^, ^22^ Prothrombotic states may cause or contribute to sino‐venous thrombosis in both adults and children. In children with sino‐venous thrombosis, the frequency of prothrombotic disorders is 12%–50%, and the presence of anticardiolipin antibody is the most common acquired disorder.^23^, ^24^ It is undetermined whether abnormal levels of prothrombotic factors, in the setting of acute treatable infections and CSVT in healthy children, are coincidental versus causal in nature.^25^ Our patient had a within the normal limit evaluation for thrombophilia, including a negative anticardiolipin antibody, Factor V Leiden PCR, antithrombin III activity, and protein C activity.

Diagnosis of typical cases of CSD can be made by a reported exposure to a cat, compatible clinical findings, and confirmation by serological tests.^26^ *Bartonella species* may require 1–4 weeks of the incubation period to appear on serological testing. *B. henselae* is usually not viable in lymph nodes of patients with CSD, so culture is not routinely used to make the diagnosis.^20^, ^27^ Pathological examination of infected tissues in immunocompetent patients, if available, will demonstrate necrotizing granulomas; the organism may be seen with a Warthin‐Starry silver stain or detected with tissue PCR. Laboratory confirmation of the clinical diagnosis is based on results of serologic testing or PCR. The high sensitivity and specificity provided by serology can explain its widespread application in clinical practice.^28^, ^29^, ^30^ The most frequently used serologic methods are indirect fluorescence assay (IFA) and enzyme immunoassay (EIA).^20^ Positive IgM titer strongly suggests acute disease, but IgM production is usually brief. IgG titers usually indicate current or recent *Bartonella* infection, even if sensitivity appears suboptimal and the prevalence of positive Bartonella serology in the general population is 4%–6%, creating false positive tests.^28^, ^30^ In our patient, semi‐quantitative IFA was done, and we observed IgM become negative as IgG titer decreased after 7 weeks from previous serology.

Polymerase chain reaction (PCR) assay involves amplification *of Bartonella species* genes (16S rRNA gene, citrate synthase gene (gltA), and htrA gene) directly from tissue, aspirate, or blood. *B. henselae* PCR tests sensitivities vary from 43% to 100%; false‐negative PCR may be explained by the lack of sensitivity, timing of sample was obtained, presence of anti‐coagulants and other PCR‐inhibitory components, and samples taken after long periods of antibiotic therapy.^31^ In our patient, with long‐standing cat exposure, the duration of illness may have exceeded 6 weeks; in addition, blood PCR was obtained after three courses of antibiotic therapy, so that a negative *B. henselae* PCR does not exclude the presence of CSD in her case, especially in the setting of a compatible clinical picture and positive serology.^32^ A PCR testing of the lymph node was not performed in our case since it is an invasive procedure and often requires sedation in pediatric patients.

Similar to previous reports, T2‐weighted, fluid‐attenuated inversion recovery, and T‐1 weighted post‐contrast‐enhanced sequences demonstrated increased signals in CSD osteomyelitis.^33^, ^34^ Diffuse or nodular osteolytic lesions have also been described in the literature. Unlike earlier reports,^8^, ^35^ no osteolytic lesions were observed in our patient, likely due to early recognition of the disease and wide availability of imaging methods such as MRI.

The value of antimicrobial therapy to treat CSD lymphadenitis is questionable and has been considered unnecessary in many healthy children.^36^ In the only controlled trial of antibiotic treatment for CSD lymphadenitis, azithromycin was found to be effective in decreasing the lymph node volume within the first 30 days of treatment.^37^

To date, only two randomized clinical trials have been reported for the treatment decisions for infections caused by *Bartonella* spp.; one of these studies evaluated patients with CSD^37^ and the other study evaluated adult patients with chronic bacteremia caused by *B. quintana*.^38^ No data are available regarding the benefits of specific antimicrobial therapy for immunocompetent patients with atypical presentations of CSD. The role of antibiotics in the improvement in disseminated disease is mainly derived from retrospective reviews. For example, a retrospective review of 202 pediatric patients with hepatosplenic CSD, suggested that rifampin, ciprofloxacin, gentamicin, and TMP‐SMX were the most efficacious antibiotics.^39^

Due to the rarity of *B. henselae* bone infection, no randomized, prospective controlled trials have been performed to establish a preferred antibiotic regimen; treatment is still controversial, and no evidence‐based guidelines are available. The large variability in antibiotic types and duration of therapy to treat *B. henselae* bone infection among studies preclude the determination of the best antibiotic regimen and length of treatment. Various antibiotic regimens, most including agents with in vitro activity against Bartonella species, have been prescribed in patients with CSD bone infection: macrolides, rifampin, beta‐lactams, TMP‐SMX, aminoglycosides, quinolones, clindamycin, and tetracyclines. Only gentamicin and rifampin appear bactericidal.^40^, ^41^ In most cases, combination or sequential use of antibiotics were used in many studies.^11^ Donà and colleagues^11^ literature review of 51 children with *B. henselae* osteomyelitis was unclear about whether the combination of rifampin and TMP‐SMX was more effective than azithromycin alone or the lesion would have healed regardless of the antibiotic therapy; in this study, 11 children who received neither medical therapy nor surgery had good prognoses.^11^ It is noteworthy that despite the use of multiple antibiotics, the clinical course of *B.henselae* infection may still be complicated by skull osteomyelitis.^42^

The length of therapy for CSD osteomyelitis remains unclear and current practice is based on case reports, small series, and personal experience. In a literature review of 62 cases of *B. henselae* osteomyelitis in children published since 1954, the length of treatment varied from 5 to 99 days (median: 22 days).^43^ Atypical manifestations of CSD, including osteomyelitis, generally have a self‐limited nature, and good prognosis.

## 4 CONCLUSION

*Bartonella* spp. are responsible for acute and chronic diseases and vascular manifestations. It is necessary to consider *Bartonella* bone spread in CSD cases with osteoarticular pain, even when there are no other signs or symptoms of systemic dissemination; infection of the skull bones associated with intra‐cranial complications should be considered in cases where persistent headaches, emesis, and other neurological symptoms are present.

Currently, serology is the gold standard for diagnosis; PCR testing of peripheral lymph nodes and other tissues is also available. MRI is the best imaging modality to define early lesions and spreading of infection to other tissues without radiation exposure. Despite its wide use, antibiotic therapy to treat CSD bone infection and other infection manifestations remain controversial; further studies are warranted to determine which patients would benefit the most from this therapeutic modality.

## AUTHOR CONTRIBUTIONS

**Ayodeji Otufowora:** Conceptualization; data curation; funding acquisition; methodology; supervision; writing – original draft; writing – review and editing. **Christine Lee:** Writing – original draft; writing – review and editing. **Aneeb Mohideen:** Writing – original draft; writing – review and editing. **Grace DeSena:** Writing – review and editing. **Charlene Pringle:** Conceptualization; methodology; supervision; writing – review and editing. **Freddie Guyer:** Supervision; writing – review and editing. **Priya Sharma:** Data curation; visualization; writing – review and editing. **Torrey D Baines:** Conceptualization; supervision; writing – review and editing. **Silvana Carr:** Conceptualization; methodology; supervision; writing – original draft; writing – review and editing.

## FUNDING INFORMATION

This work was supported by the Children's Miracle Network Hospitals Grant (PI: Dr Ayodeji Otufowora, FY22 CMN Award).

## CONFLICT OF INTEREST STATEMENT

The authors have no conflicts of interest to disclose.

## CONSENT

Written and informed consent was obtained from the child's mother.

## Data Availability

The data used to support the findings of this study are included within the article.
